# Prevalence and predictors of long-acting reversible contraceptive use among sexually active women in 26 sub-Saharan African countries

**DOI:** 10.1093/inthealth/ihab053

**Published:** 2021-08-18

**Authors:** Obasanjo Afolabi Bolarinwa, Ugochinyere Ijeoma Nwagbara, Joshua Okyere, Bright Opoku Ahinkorah, Abdul-Aziz Seidu, Edward Kwabena Ameyaw, Victor Igharo

**Affiliations:** Department of Public Health Medicine, School of Nursing and Public Health, University of KwaZulu-Natal, Durban 4041, South Africa; Department of Public Health Medicine, School of Nursing and Public Health, University of KwaZulu-Natal, Durban 4041, South Africa; Department of Population and Health, University of Cape Coast, Cape Coast,PMB, Ghana; School of Public Health, University of Technology, Sydney, NSW 2007, Australia; Department of Population and Health, University of Cape Coast, Cape Coast,PMB, Ghana; College of Public Health, Medical and Veterinary Services, James Cook University, Townsville, Queensland, QLD4811, Australia; Department of Estate Management, Takoradi Technical University, P.O. Box 256, Takoradi, Ghana; School of Public Health, University of Technology, Sydney, NSW 2007, Australia; John’s Hopkins Centre for Communications Programs, 111 Market Place Suite 310 Baltimore, MD, USA

**Keywords:** contraceptive, long-acting reversible, predictors, public health, sexually active women, sub-Saharan African

## Abstract

**Background:**

Long-acting reversible contraceptives (LARCs) are associated with high efficacy rates and continuity of use. Based on the foregoing, we sought to examine the prevalence and factors associated with LARC use among sexually active women in 26 countries in sub-Saharan Africa(SSA).

**Methods:**

Secondary data from Demographic and Health Surveys conducted in 26 countries in SSA between January 2010 and December 2019 were pooled and analysed. A total of 56 067 sexually active women 15–49 y of age met the inclusion criteria. Bivariate and multivariate regression analyses were performed to examine the association between selected factors and the use of LARCs in SSA. Results were presented as crude odds ratios and adjusted odds ratios (aORs) with statistical precision at <0.05.

**Results:**

The prevalence of LARC use was 21.73%, ranging from 1.94% in Namibia to 54.96% in Benin. Sexually active women with secondary or higher education (aOR 1.19 [95% confidence interval {CI} 1.08 to 1.32]), those cohabiting (aOR 1.25 [95% CI 1.06 to 1.47]) and those with four or more children (aOR 2.22 [95% CI 1.78 to 2.78]) were more likely to use LARCs compared with those without education, never married and with no biological child.

**Conclusions:**

The use of LARCs in the 26 countries in SSA was relatively low. Hence, the identified contributory factors of LARC use should be tackled with appropriate interventions. These include continuous campaigns on the efficacy of LARCs in reducing unintended pregnancy, maternal mortality and morbidity.

## Introduction

Globally, there have been significant efforts to reduce unintended pregnancies and fertility rates.^1,2^ One such effort has been the campaign for the use of modern contraceptives among sexually active women.^3^ Available evidence shows that these actions have effectively increased contraceptive use among sexually active women.^4^ However, most sexually active women who use modern contraceptives use short-term methods such as oral contraceptive pills and condoms, which have been identified as being significantly associated with high rates of discontinuation.^5–7^ This situation has persisted at the expense of long-acting reversible contraceptives (LARCs).

LARCs may be viewed as hormonal and copper intrauterine devices and contraceptive implants.^8^ Globally not more than 15% of women use LARCs.^9^ Europe, for instance, has a varied prevalence of LARC use; the prevalence of LARC use in Poland is 2.9%, while France has a prevalence of about 16%, which is higher than the global proportion of women using LARCs.^10^ Other regions, such as Latin America and Asia, have recorded a high prevalence of LARC use.^11,12^ However, in sub-Saharan Africa (SSA), the prevalence of LARC use among women is <3%.^13^

LARCs are known to have fewer contraindications and are without age restrictions.^14^ Moreover, they are cost effective and highly efficacious in preventing unintended pregnancy.^15–17^ Given that LARC use is independent of user compliance, they are more efficacious, with an annual pregnancy rate of <1% for women using LARCs compared with their counterparts who rely on short-term contraceptives such as oral pills and condoms, which have annual pregnancy rates of 9% and 18%, respectively.^8^ Thus LARCs are a more efficacious choice.

Nevertheless, the availability and use of LARCs remains low among sexually active women, especially those living in SSA.^1,13,18^ Studies show that there may be some potential adverse health concerns with the use of LARCs, which serves as a disincentive for many women to accept and utilize them.^19^ LARCs are associated with multiple adverse health events, including distortion of menstrual bleeding (either heavy or less than usual bleeding), an increase in the risk of iron deficiency anaemia and pelvic pain.^19–21^ Other factors such as the availability of LARCs, misconceptions about their use and the perceived cost are some of the barriers to the use of LARCs.^22^ This raises concerns about how demographic (age, place of residence, etc.) and socio-economic factors (such as wealth quintile, educational level, media exposure) affect LARC use among sexually active women.

Given the importance of LARCs in ensuring high efficacy rates and contraceptive continuation,^23^ the need to understand the factors that predict their use among sexually active women has become paramount to the realization of better reproductive health outcomes and higher uptake of LARCs. Despite this urgency for a better understanding of the predictors of LARCs, it has received little attention in SSA. Therefore, we sought to bridge this literature gap by examining the prevalence and predictors of LARC use among sexually active women in SSA using the most recent Demographic and Health Survey (DHS) data from 26 countries. We hope this study's findings will be a contributing catalyst to Africa's efforts to reduce maternal mortality and morbidity and significantly reduce unintended pregnancies linked to Sustainable Development Goal 3 by increasing the coverage and use of LARCs.

## Methods

### Data source and study design

Data utilized in this study were pooled from DHSs conducted in 26 countries in SSA between 2010 and 2019. The countries, years the survey was conducted and sample size for each country are listed in Table 1. The DHS dataset is a nationally representative survey conducted in about 85 low- and-middle-income countries with a core target of sexual and reproductive health issues, including contraceptive use.^24^ The women's files, which contain information on women of reproductive age, were used for this study.

**Table 1.** Sample size distribution by country and survey year

Survey countries	Survey year	Weighted sample	Weighted percentage
Central Africa			
Democratic Republic of Congo	2014	1034	1.84
Cameroon	2019	1476	2.63
Chad	2015	497	0.89
Malawi	2016	1327	2.37
Rwanda	2015	3190	5.69
West Africa			
Burkina Faso	2010	1878	3.35
Benin	2018	1241	2.21
Gambia	2013	457	0.82
Guinea	2018	602	1.07
Liberia	2013	1374	2.45
Nigeria	2018	3100	5.53
Niger	2012	753	1.34
Mali	2018	1327	2.37
Sierra Leone	2013	2473	4.41
Senegal	2018	1267	2.26
Togo	2014	1075	1.92
Ghana	2014	1133	2.02
East Africa			
Kenya	2014	4405	7.86
Ethiopia	2016	1132	5.92
Tanzania	2016	2738	4.88
Uganda	2011	3639	6.49
Southern Africa			
Comoros	2012	380	0.68
Lesotho	2014	1774	3.16
Namibia	2013	2337	4.17
South Africa	2016	2484	4.43
Zimbabwe	2018	3720	6.63

Source: DHS from 26 SSA countries: 2010–2019.

The DHS employed stratified two-stage sampling techniques to ensure national representativeness.^25^ The first stage was the development of a sampling frame containing a list of primary sampling units (PSUs), also known as enumeration areas (EAs), which cover each participating country's entirety. The PSUs are obtained from the latest available national census. Each PSU is further subdivided into standard size segments of about 100–500 households per segment. Random selection of predetermined sample segments is done afterward with probability proportional to the EA's measure of size depending on the number of households in each EA.

The second stage entails the systematic selection of households from the list of households previously enumerated by DHS personnel. Women 15–49 y of age and men 15–64 y of age within the selected households are then interviewed. The countries included in the present study had their surveys conducted at different times due to variations in the starting points of DHS data collection in these countries. Nomads and persons in institutional groups, such as prisoners and hotel occupants, are usually excluded from the sample frame. The use of multiple DHS datasets from SSA countries has been previously achieved in other studies.^26,27^ Despite the variation in starting points of surveys in various countries, we compared the DHSs among the countries. Permission to utilize the 26 SSA datasets was sought from Monitoring and Evaluation to Assess and Use Results (MEASURE) DHS and the datasets are freely available publicly at https://dhsprogram.com/data/available-datasets.cfm.

### Sample size and inclusion criteria

A total of 56 067 women 15–49 y of age who were sexually active (those who had ever had sex) and were using modern contraceptives at the time of the surveys were included in our study; a total of 26 SSA countries were included in the final analysis. These records were used for the analysis because they had complete sets of all the variables of interest in the current study.

### Definition of study variables

#### Outcome variable

The study's outcome variable was ‘LARC,’ which arose from the question about the type of contraceptive women 15–49 y of age who were sexually active were using at the time of the survey. Women who were using modern contraceptives such as condoms, pills, injections, female sterilization, vasectomy, emergency contraceptives and other methods were categorized as not using LARC, while those using an intrauterine device (IUD) or implant were categorized as using LARC.^13,28^ Those using methods other than modern contraceptives were excluded from the study.

#### Explanatory variables

Nine explanatory variables were considered in this study, including age, place of residence, education level, marital status, currently working, wealth index, parity, media exposure and the desire for more children. These variables have been reported to predict LARC use in previous nationally representative studies.^28–30^ Four of the explanatory variables were recoded for better analysis outputs and interpretation. The age of respondents was recoded as 15–24 (0), 25–34 (1) and ≥35 (2). Marital status was recoded into never married (0), married (1), cohabiting (2) and ever married (3). We recoded parity (children ever born) as zero births (0), one to three births (1) and four or more births (2). Finally, exposure to mass media was recoded into no (0) for those who do not read the newspaper, listen to the radio or watch television and yes (1) for those who read the newspaper, listen to the radio or watch television at least once a week.

### Statistical analyses

The analysis began with the computation of LARC use among sexually active women of reproductive age in 26 countries in SSA. We then appended the dataset from the countries, generating a total sample of 56 067. We calculated the overall prevalence, proportions of LARC use and chi-square values across the selected sociodemographic characteristics after appending. Binary logistic regression analysis was performed in two models: the first model (model 1) was a bivariate analysis of the country effect on LARC use. The reference country was Angola, due to the limited contraceptive use that has been reported in that country.^31^ Model 2 adjusted for the effects of the other explanatory variables to ascertain how these variables influence LARC use. The choice of reference categories for the explanatory variables was determined by similar previous studies.^30,32^ Binary logistic regression modelling was employed because our dependent variable (LARC use) was measured as a binary factor. We presented the regression analysis results as crude odds ratios (cORs) and adjusted odds ratio (aORs), with their corresponding 95% confidence intervals (CIs) signifying the precision and significance of the reported OR. The multicollinearity test, which used the variance inflation factor (VIF), revealed no collinearity among the independent variables employed in this study (mean VIF 1.44, maximum VIF 1.96 and minimum VIF 1.06). The inherent survey sample weight to control for undersampling and non-responses was applied. All analyses were carried out with Stata version 16.0 (StataCorp, College Station, TX, USA).

## Results

### Descriptive results

**Figure 1.**
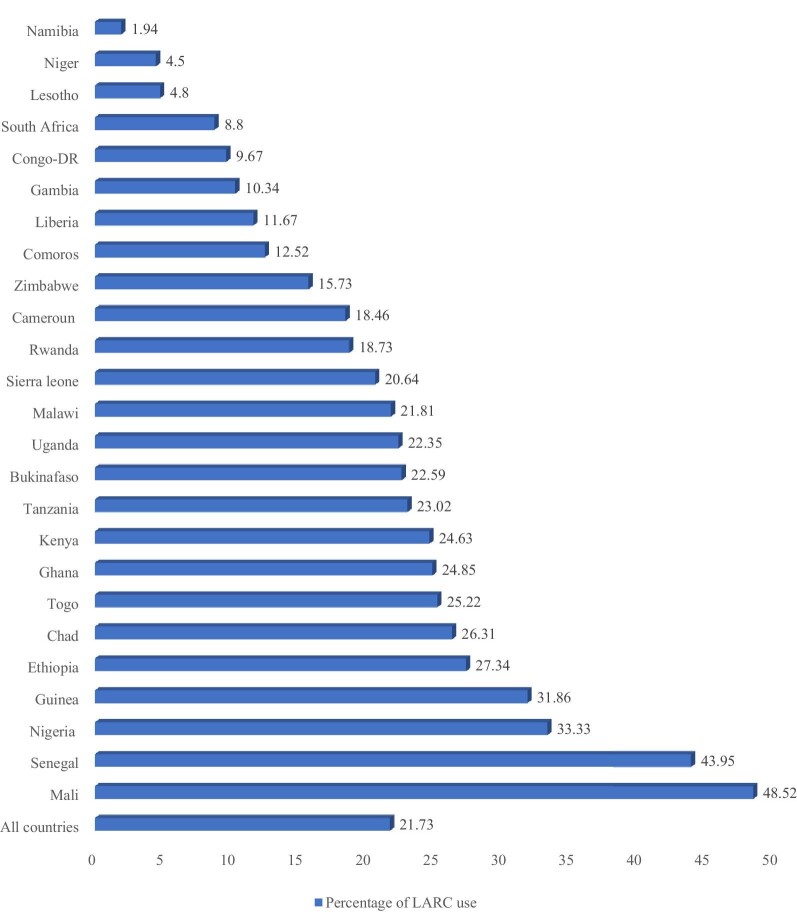
Prevalence of LARC use in SSA among sexually active women.

Figure 1 presents the prevalence of LARC use in each of the 26 SSA countries included in the study. The prevalence of LARC use ranged from 1.94% in Namibia to 54.96% in Benin. Overall, the prevalence of LARC use among sexually active women in the 26 SSA countries was 21.73%.

Table 2 shows results of the distribution of sexually active women currently using modern contraceptives in SSA by explanatory variable. The results show a high prevalence of LARC use among sexually active women ages 25–34 y (23.74%), those residing in a rural area (37.91%), those with no education (26.90%), ever married sexually active women (24.14%), women currently employed (23.13%), those within the richest wealth index quintile (23.45%), those with four or more children (24.91%), sexually active women not exposed to mass media (22.00%) and those who were undecided if they desire more children (26.15%). All selected explanatory variables were significant at 0.001, except the place of residence and mass media exposure, with p-values >0.05.

**Table 2.** Distribution of sexually active women using LARCs by explanatory variables in SSA (N=56 067)

			Using LARCs, n (%)		
Variables	Weighted Frequency (n)	Weighted Percentage	No	Yes	p-Value (χ^2^)
Age (years)					<0.001
15–24	13 961	24.90	10 937 (81.80)	2433 (18.20)	
25–34	24 092	42.97	17 595 (76.26)	5478 (23.74)	
35–49	18 015	32.13	13 494 (78.21)	3759 (21.79)	
Place of residence					0.98
Urban	23 312	41.58	17 476 (78.28)	4850 (21.72)	
Rural	32 755	58.42	24 550 (78.26)	6820 (21.74)	
Education level					<0.001
No education	11 015	77.68	7712 (73.10)	2838 (26.90)	
Primary	21 685	22.32	16 365 (78.80)	4403 (21.20)	
Secondary/higher	23 367		17 950 (80.21)	4428 (19.79)	
Marital status					<0.001
Never married	6567	11.71	5566 (88.51)	722 (11.49)	
Married	39 725	70.85	28 860 (75.86)	9185 (24.14)	
Cohabiting	7653	13.65	5942 (81.08)	1386 (18.49)	
Ever married	2123	3.79	1657 (81.51)	375 (21.73)	
Currently working					<0.001
No	18 381	32.78	14 281 (81.12)	3323 (18.88)	
Yes	37 686	67.22	27 746 (76.87)	8347 (23.13)	
Wealth index					<0.001
Poorest	6916	12.34	5226 (78.90)	1398 (21.10)	
Poorer	9415	16.79	7186 (79.69)	1831 (20.31)	
Middle	10 531	18.78	7935 (78.67)	2151 (21.33)	
Richer	13 163	23.48	9919 (78.68)	2688 (21.32)	
Richest	16 042	28.61	11 761(76.55)	3602 (23.45)	
Parity					<0.001
No children	4448	7.93	3782 (88.78)	478 (11.22)	
1–3 children	29 039	51.79	22 004 (79.12)	5806 (20.88)	
≥4 children	22 581	40.27	16 240 (75.09)	5386 (24.91)	
Media exposure					0.57
No	11 644	20.77	8698 (78.00)	2453 (22.00)	
Yes	44 424	79.23	33 328 (78.34)	9217 (21.66)	
Desire for more children					<0.001
Want more	30 632	54.63	23 119 (78.80)	6218 (21.20)	
Undecided	2068	3.69	1462 (73.85)	518 (26.15)	
No desire	23 367	41.68	17 445 (77.95)	4934 (22.05)	

### Association between selected explanatory variables and LARC use among sexually active women in SSA

Two models were fitted to examine the association between selected explanatory variables and LARC use, with the results presented in Table 3. Model 1 was a crude model that was unadjusted and model 2 adjusted for the confounders. In model 2, a statistically significant effect of LARC use was found in some selected explanatory variables and countries in SSA.

**Table 3.** Bivariate and multivariable models showing the relationship between LARC use and selected explanatory variables among sexually active women in SSA countries

	Model 1 (unadjusted)		Model 2 (adjusted)	
Variables	cOR	95% CI	aOR	95% CI
Age (years)				
15–24	Ref	Ref		
25–34	1.40^***^	1.31–1.50	1.08	1.00 to 1.17
≥35	1.25^***^	1.16 to 1.35	0.85^**^	0.77 to 0.95
Place of residence				
Urban	Ref	Ref		
Rural	1.00	0.94 to 1.07	0.93	0.85 to 1.01
Education level				
No education	Ref	Ref		
Primary	0.73^***^	0.68 to 0.79	1.02	0.94 to 1.11
Secondary/higher	0.67^***^	0.62 to 0.73	1.19^***^	1.08 to 1.32
Marital status				
Never married	Ref	Ref		
Married	2.45^***^	2.20 to 2.73	1.38^***^	1.17 to 1.63
Cohabiting	1.80^***^	1.59 to 2.03	1.25^**^	1.06 to 1.47
Ever married	1.75^***^	1.48 to 2.07	1.18	0.97 to 1.44
Currently working				
No	Ref	Ref		
Yes	1.29^***^	1.22 to 1.37	1.06	1.00 to 1.13
Wealth index				
Poorest	Ref	Ref		
Poorer	0.95	0.87 to 1.04	0.93	0.85 to 1.01
Middle	1.01	0.92 to 1.11	0.96	0.88 to 1.06
Richer	1.01	0.92 to 1.11	0.95	0.85 to 1.04
Richest	1.15^**^	1.04 to 1.26	1.03	0.92 to 1.16
Parity				
No children	Ref	Ref		
1–3 children	2.09^***^	1.80 to 2.42	1.94^***^	1.58 to 2.38
≥4 children	2.63^***^	2.27 to 3.04	2.22^***^	1.78 to 2.78
Media exposure				
No	Ref	Ref		
Yes	0.98	0.92 to 1.05	1.03	0.96 to 1.11
Desire for more children				
Want more	Ref	Ref		
Undecided	1.32^***^	1.15 to 1.51	1.21^**^	1.05 to 1.39
No desire	1.05	0.99 to 1.11	1.12^**^	1.05 to 1.21
Country				
Democratic Republic of Congo	Ref	Ref		
Cameroon	4.18^***^	3.47 to 5.04	4.30^***^	3.55 to 5.19
Chad	0.37^***^	0.24 to 0.55	0.36^***^	0.24 to 0.54
Malawi	0.78^*^	0.64 to 0.95	0.81^*^	0.66 to 0.99
Rwanda	1.29^*^	1.05 to 1.59	1.31^*^	1.06 to 1.62
Burkina Faso	1.13	0.90 to 1.43	1.11	0.87 to 1.41
Benin	0.40^***^	0.25 to 0.62	0.35^***^	0.23 to 0.54
Gambia	1.60^***^	1.26 to 2.04	2.02^***^	1.55 to 2.63
Guinea	1.12	0.95 to 1.32	1.02	0.86 to 1.21
Liberia	0.49^**^	0.32 to 0.74	0.48^**^	0.32 to 0.73
Nigeria	0.45^***^	0.32 to 0.64	0.49^***^	0.35 to 0.70
Niger	0.17^***^	0.13 to 0.23	0.16^***^	0.12 to 0.22
Mali	3.23^***^	2.62 to 3.98	3.15^***^	2.55 to 3.88
Sierra Leone	0.96	0.82 to 1.12	0.90	0.76 to 1.05
Senegal	1.71^***^	1.44 to 2.05	1.55^***^	1.29 to 1.87

**Table 3.** Continued.

	Model 1 (unadjusted)		Model 2 (adjusted)	
Variables	cOR	95% CI	aOR	95% CI
Togo	0.16^***^	0.11 to 0.24	0.15^***^	0.10 to 0.23
Ghana	0.07^***^	0.05 to 0.99	0.07^***^	0.05 to 0.11
Kenya	0.79^**^	0.67 to 0.93	0.77^**^	0.65 to 0.91
Ethiopia	0.89	0.74 to 1.07	1.05	0.87 to 1.28
Tanzania	2.69^***^	2.08 to 3.47	2.57^***^	1.98 to 3.33
Uganda	1.22	0.88 to 1.71	1.18	0.84 to 1.65
Comoros	1.16	0.92 to 1.45	1.21	0.96 to 1.52
Lesotho	1.02	0.85 to 1.23	1.02	0.84 to 1.24
Namibia	0.99	0.84 to 1.16	0.95	0.80 to 1.13
South Africa	0.33^***^	0.26 to 0.42	0.34^***^	0.26 to 0.44
Zimbabwe	0.64^***^	0.54 to 0.76	0.56^***^	0.47 to 0.68
Pseudo-R^2^			0.0772	

Model 1: unadjusted model examining the association of selected factors and LARC use. Model 2: adjusted for confounders. Exponentiated coefficients. cOR, Crude Odds Ratio; AOR, Adjusted Odds Ratio; CI, Confidence Interval.

Ref: reference.

^*^p<0.05, ^**^p <0.01, ^***^p<0.001.

The likelihood of using LARCs was higher among sexually active women with a secondary or higher education (aOR 1.19 [95% CI 1.08 to 1.32]), those who were married (aOR 1.38 [95% CI 1.17 to 1.63]), those cohabiting (aOR 1.25 (95% CI 1.06 to 1.47]), sexually active women with one to three children (aOR 1.94 [95% CI 1.58 to 2.38]) and those with four or more children (aOR 2.22 [95% CI 1.78 to 2.78]) compared with those without education, those ever married and those with no children, respectively, while those ≥35 y of age were less like to use LARCs (aOR 0.85 [95% CI 0.77 to 0.95]).

SSA countries with lower odds of using LARCs in the adjusted model (model 2) included Chad (aOR 0.36 [95% CI 0.24 to 0.54]), Benin (aOR 0.35 [95% CI 0.23 to 0.54]), Nigeria (aOR 0.49 [95% CI 0.35 to 0.70]), Ghana (aOR 0.07 [95% CI 0.05 to 0.11]) and South Africa (aOR 0.34 [95% CI 0.26 to 0.44]), while sexually active women in Cameroon (aOR 4.30 [95% CI 3.55 to 5.19]) had higher odds of using LARCs than any other SSA countries (see Table 3).

## Discussion

LARCs may help alleviate the high unmet need for modern contraception in SSA, as they are more cost effective and efficacious and not reliant on user compliance.^33,34^ Yet empirical evidence on this is sparse in SSA. This study examined the prevalence and predictors of LARC use among sexually active women using modern contraceptives in 26 SSA countries. The prevalence of LARC use ranged from 4.50% in Niger to 54.96% in Benin and overall the prevalence of LARC use among sexually active women in the 26 SSA countries was 21.73%. This result is higher than in a study conducted among women in SSA that reported LARC use of <3%.^13^

Our study found that the likelihood of using LARCs was higher among sexually active women with secondary or higher education compared with those without education. This is because most educated women have increased access to information on the side effects and benefits of using LARC methods.^35^ As such, they are aware of the misconceptions and myths that often serve as a deterrent to the use of LARCs. Studies conducted in Kenya,^34^ Ethiopia^35,36^ and Uganda^37^ have shown that higher education is an important predictor of LARC use.

This study showed that the prevalence of contraceptive use was higher among sexually active women who were married compared with those who were ever married. This finding is supported by several studies that have demonstrated a positive correlation between marriage and modern contraceptive use.^38,39^ However, this contradicts a study conducted in the Democratic Republic of Congo that reported that modern methods and LARC use were lower among married women.^40^ This could be related to family or social pressure to give birth quickly after marriage.^40^ Therefore there is a need to introduce contraception use among sexually active women during antenatal care visits to encourage child spacing and delay subsequent pregnancy.

In this study, sexually active women cohabiting with their partners were more likely to use LARCs. This could be to avoid being exposed to unintended pregnancy, thus leaving them with no choice but to have a positive attitude towards LARC methods.^41^ The study also showed that sexually active women with one to three children and those with four or more children had a similar likelihood of using LARCs compared with those with no children. As the number of children increased, the likelihood of using LARCs also increased.^42^ A possible explanation for this finding could be that multiparous women tend to receive family planning education and counselling on contraceptive use throughout their pregnancy cycle, thereby increasing their odds of using LARCs.^42^ This corroborates findings from other studies where marital status and parity have been identified as important individual characteristics influencing women's reproductive health behaviours, including demand and uptake of modern contraception.^43–45^

Also, sexually active women who were ≥35 y of age were less likely to use LARCs compared with younger women. This is contrary to a study conducted in Kenya that showed more LARC usage among those ages 20–24 y.^46^ Increasing LARC usage in SSA countries will require continuous community awareness campaigns to counteract negative perceptions and misinformation.^47^

Our study revealed that sexually active women from some SSA countries such as Chad, Benin, Nigeria, Ghana and South Africa had lower odds of using LARCs, while women in Cameroon had higher odds of using LARCs than in any other SSA countries. This may be because there are programmes in place that encourage LARC uptake and this is more markedly documented among sexually active women in Cameroon.^48^

The positive efforts of the Cameroon government and family planning providers to increase modern contraceptive practice has ensured higher usage of LARCs.^49^ The findings of this study will help policymakers, governments and family planning providers to inform policy regarding the benefits of using LARCs and address the various barriers to the uptake of LARC methods in SSA.

### Strengths and limitations

The large sample size from countries in SSA is the major strength of this study, making the findings applicable to sexually active women in SSA. A limitation of this study is that it is a cross-sectional survey, so the progressive order of contraceptive use. The study employed a cross-sectional design, therefore, we could only measure associative effects but not causal effect. The different interventions recommended in this study may not be applicable in all countries in SSA, as economic, cultural and social differences between SSA countries may influence LARC usage. Furthermore, this study analysis did not consider the effects of accessibility to LARCs and women's beliefs related to LARC use.

### Policy implications for SSA countries

The findings of this study hold some policy implications for countries in SSA. Generally, women with a secondary or higher education were more likely to use LARCs, which shows the need to target women without education and enlighten them on the benefits of LARCs use. Family planning providers need to counsel women without children concerning their fears about side effects and misconceptions of LARCs and emphasize that LARC methods are reversible.

## Conclusions

We found that the use of LARCs in the 26 countries in SSA considered in this study is relatively low. There is a need for sexually active women in SSA to receive counselling on the benefits of using LARCs. Furthermore, governments, policymakers and stakeholders should create awareness by implementing health promotion strategies to increase the need for LARC usage among sexually active women in SSA. Implementation of educational campaigns for sexually active women with lower education should be prioritized in SSA countries. Also, intervention programmes such as campaigns on the efficacy of LARCs in reducing unintended pregnancy and maternal mortality and morbidity should be encouraged among sexually active women with low LARC use.

## Data Availability

The datasets for all the countries can be accessed at https://dhsprogram.com/data/available-datasets.cfm.
